# Treatment of Tularemia in Patient with Chronic Graft-versus-Host Disease

**DOI:** 10.3201/eid1905.120377

**Published:** 2013-05

**Authors:** Jan Weile, Erik Seibold, Cornelius Knabbe, Martin Kaufmann, Wolf Splettstoesser

**Affiliations:** Heart and Diabetes Centre North Rhine–Westphalia, Bad Oeynhausen, Germany (J. Weile, C. Knabbe);; Bundeswehr Institute of Microbiology, Munich, Germany (E. Seibold, W. Splettstoesser);; University of Rostock, Rostock, Germany (W. Splettstoesser);; Robert-Bosch-Hospital, Stuttgart, Germany (M. Kaufmann)

**Keywords:** Francisella tularensis ssp. holarctica, chronic graft-versus-host disease, immunocompromised host, vector-borne infections, bacteria, tularemia

## Abstract

We describe a case of human tularemia caused by *Francisella tularensis* subsp. *holarctica* in a stem cell transplant recipient with chronic graft-versus-host disease who was receiving levofloxacin prophylaxis. The infection was characterized by pneumonia with septic complications. The patient was successfully treated with doxycycline.

Tularemia is a zoonotic infection caused by the gram-negative bacterium *Francisella tularensis*. Humans are accidental hosts; infection occurs after contact with infected animals, contaminated water or soil, or invertebrate vectors (*1*). Strains of the 2 subspecies F. *tularensis* subsp. *tularensis* and F. *tularensis* subsp. *holarctica* account for virtually all infections in humans. Only rarely have strains of the subspecies *F. tularensis novicida* or the closely related species *F. philomiragia* or *F. hispaniensis* been cultured from clinical specimens (*2*).

*F. tularensis* subsp. *tularensis*, also referred to as type A, is found almost exclusively in North America and is the most virulent subspecies. *F. tularensis* subsp. *holarctica*, also referred to as type B, is found predominantly in Asia and Europe, but also in North America (*3*). Patients infected with *F. tularensis* have abrupt onset of fever, chills, headache, and malaise after an incubation period of 2–21 days. Additional signs and symptoms may develop, depending on the portal of entry. The most common signs and symptoms are lymphadenopathy, fever, pharyngitis, appearance of ulcers/eschars/papules, nausea and vomiting, and hepatosplenomegaly.

Antimicrobial drug therapy should be administered to patients with this suspected or confirmed diagnosis, even though spontaneous resolution may occur in 50%–95% of cases (depending on the clinical syndrome) (*4*). For severe tularemia, gentamicin is the drug of choice (5 mg/kg/d, divided into 2 doses and monitored by analysis of serum drug concentration). If available, streptomycin is a well-suited alternative agent. This approach is based on observational data evaluating frequency of cure and relapse with different antimicrobial drugs (*5*) and is currently recommended by the World Health Organization (*6*). Oral agents may be used for treatment of mild illness. Preferred agents are doxycycline or ciprofloxacin. Observational data for tetracycline have found an 88% cure rate and 12% relapse rate (*5*), although other studies have indicated that relapse might be more common in patients who received tetracycline than in those who received ciprofloxacin or aminoglycosides (*6*).

##  The Study

A 54-year-old man was admitted to the hematology department, Robert-Bosch-Hospital, Stuttgart, Germany, in early 2010, with fever (39.5°C), chills, and minor dyspnea that had lasted for 3 days. Four years earlier, he had received a stem cell transplant for acute myeloid leukemia, which was in first complete remission after myeloablative conditioning with total body irradiation, 12 Gy, and cyclophophamide, 120 mg/kg bodyweight. The post-transplant course was complicated by grade 3 graft-versus-host-disease of the skin and gut, multiple infectious episodes, chronic renal failure (creatinine level 3 mg/dL, glomerular filtration rate 25 mL/min, urea level 80 mg/dL), repeatedly occurring cytomegalovirus replications, and later on extensive chronic graft-versus-host-disease, necessitating continuous immunosuppressive therapy (tacrolimus, steroids), and anti-infective prophylaxis (dose-adjusted levofloxacin, 125 mg/d, posaconazole, 3 × 200 mg/d), respectively. After admission, chest radiograph revealed no abnormal findings. Blood cultures were drawn, and the patient was given empiric antimicrobial drug therapy with intravenous (IV) imipenem/cilastatin (500 mg/8 h) and full-dose levofloxacin (IV 2 × 250 mg/d) the same day.

Fever persisted, and a computerized tomography scan and a bronchoscopy were performed. The computerized tomography scan revealed a large infiltrate in the right upper lobe. In the initially drawn blood cultures, gram-negative rods were cultivated after 165 h. *F. tularensis* was suspected on the basis of biochemical identification, and the isolate was sent to the reference laboratory for tularemia for confirmation (Bundeswehr Institute of Microbiology, Munich, Germany). Subsequently (day 8 after admission), the antimicrobial drug therapy was extended to doxycycline (IV 2 ×100 mg/d). Aminoglycoside therapy was avoided because of the chronic renal failure. The patient was discharged afebrile after 16 days in improved condition.

Examination of smears originating from the positive blood cultures revealed bacteria that presented as pleomorphic, faintly staining, gram-negative coccobacilli. An aerobic, slow-growing bacterium was recovered from chocolate agar after a 2-day incubation at 37°C in an atmosphere of 5% CO_2_. Presumptive identification of the isolate with the gram-negative card on the VITEK 2XL instrument (bioMérieux, Nörtingen, Germany) indicated *F. tularensis.* The isolate was sent to the national reference laboratory for tularemia for confirmation and further characterization.

The presumptive phenotypic identification of the *F. tularensis* strain was confirmed by real-time PCR that targeted *Francisella*-specific 16S rDNA sequences (*Francisella* LightMix kit; TIP MOLBIOL, Berlin, Germany), a type B–specific real-time PCR that targeted the 23S rDNA gene, as well as 23S rDNA sequencing. Molecular analysis of the 23S rDNA sequence and phenotypic determination of macrolides susceptibility (Etest; bioMérieux) revealed that the isolate represented a strain of *F. tularensis holarctica* biovar I.

Antimicrobial drug susceptibility testing was performed according to the current recommendations of the Clinical and Laboratory Standards Institute (*7*) by using the commercially available, CE-certified MICRONAUT-S-microtiter broth dilution testing system (Merlin, Bornheim, Germany) and gave results characteristic for all *F. tularensis* strains (Table). Although the strain was susceptible for levofloxacin, according to Clinical and Laboratory Standards Institute standards, the MIC of 0.25 mg/L was at least twice as high when compared with 69 other *F. tularensis* subsp. *holarctica* strains (0.031, n = 11; 0.062, n = 54; 0.125, n = 4) (*7*). Multilocus variable number of tandem repeats analysis (*8*) demonstrated that the strain clustered with 10 additional German *F. tularensis* strains from which 3 were isolated from hares found in an area <20 km from the patient’s home.

**Table:** Susceptibility testing of the blood culture isolate (*Francisella tularensis* subsp. *holarctica*) break point analysis according to CLSI standard*

Antimicrobial agent	MIC, mg/L	Interpretation
Tetracycline	1.0	S
Ciprofloxacin	0.125	S
Levofloxacin	0.25	S
Chloramphenicol	2	S
Gentamicin	0.5	S
Streptomycin	2	S

*CLSI, Clinical and Laboratory Standards Institute; S, susceptible; R, resistant.

## Conclusions

Because infection with *F. tularensis* or other *Francisella* species is relatively infrequent in nature, informative examples of infection in immunocompromised persons are rare. Elkins et al. provide a comprehensive review of *F. tularensis* infections in this patient collective, most of which occur in patients who have had a solid organ transplant or who have AIDS (*9*). Only 2 cases of tularemia caused by *F. tularensis* in stem cell or bone marrow transplant patients have been reported to date; 1 patient died because of severe neutropenia (*10*). 

In the patient described here, tularemia was acquired while he was undergoing prophylaxis with levofloxacin, a potential active agent against *F. tularensis*. This failure of anti-infective prophylaxis was most probably related to the reduced levofloxacin concentration caused by renal-based dose adaption rather than because the strain had developed fluoroquinolone resistance. Although the strain isolated from the blood stream of the patient was susceptible to levofloxacin by an approved microdilution broth assay, heterogeneous resistance against fluoroquinolones could not be completely ruled out for methodologic reasons.

It has been well documented that *F. tularensis* can easily be rendered ciprofloxacin-resistant because of single nucleotide polymorphisms of the quinolone-resistant determining region of gyrase A, but such strains have so far never been isolated from patients with clinical cases (*11*). Although doxycycline is not considered the drug of choice for severe tularemia, treatment with IV doxycycline for 16 days was successful for this patient. Thus, doxycycline might be a useful alternative for treating cases in which aminoglycosides or fluoroquinolones cannot be applied or may have failed.

Careful and comprehensive survey and questioning of the patient did not definitely reveal the route of infection. The patient did not recall any insect bites or contact with animals. The only potential risk factor mentioned was mowing the lawn of his garden close to a forest about 9 days before onset of symptoms. The home region of the patient is thought to be highly endemic for tularemia. Although it remains unproven, this hypothesis is consistent with the molecular epidemiologic results, demonstrating that identical or nearly identical genotypes were found near the patient’s home.
